# The role of community pharmacists in managing common headache disorders, and their integration within structured headache services: position statement on behalf of the European Headache Federation (EHF) and *Lifting The Burden* (LTB: the Global Campaign against Headache), with the formal endorsement of the International Pharmaceutical Federation

**DOI:** 10.1186/s10194-025-02021-3

**Published:** 2025-05-06

**Authors:** Heba BaniHani, Christian Lampl, Antoinette MaassenvandenBrink, Faisal Mohammad Amin, Louise Ninett Carlsen, Gianluca Coppola, Christina Deligianni, Raquel Gil-Gouveia, Philip R. Holland, Andreas K. Husøy, Rigmor Jensen, Madalena Plácido, Uwe Reuter, Kristina Ryliškienė, Margarita Sanchez del Río, Henrik Winther Schytz, Erling Tronvik, Jan Versijpt, Timothy J. Steiner

**Affiliations:** 1 Norwegian Centre for Headache Research (NorHead), Trondheim, Norway; 2 Department of Neurology, Konventhospital Barmherzige Brüder, Linz, Austria; 3 Headache Medical Center Linz, Linz, Austria; 4 Department of Internal Medicine, Erasmus MC University Medical Center, Rotterdam, Netherlands; 5 Department of Neurology, Danish Headache Centre, Copenhagen University Hospital - Rigshospitalet, Copenhagen, Denmark; 6 Department of Medico-Surgical Sciences and Biotechnologies, Sapienza University of Rome Polo Pontino ICOT, Latina, Italy; 7 Department of Neurology, AthensvNaval Hospital, Athens, Greece; 8 1st Neurology Department, Eginition Hospital, Medical School, National & Kapodistrian University of Athens, Athens, Greece; 9 Neurology Department, Hospital da Luz, Luz Saúde, Lisbon, Portugal; 10 Center for Interdisciplinary Research in Health, Universidade Católica Portuguesa, Lisbon, Portugal; 11 Headache Group. Wolfson Sensory Pain and Regeneration Centre, Institute of Psychiatry, Psychology and Neuroscience, King’S College London, London, UK; 12 Department of Neuromedicine and Movement Science, Norwegian University of Science and Technology (NTNU), Trondheim, Norway; 13 Department of Neurology and Clinical Neurophysiology, St Olavs University Hospital, Trondheim, Norway; 14 NOVA National School of Public Health, Public Health Research Centre, Comprehensive Health Research Centre, NOVA University of Lisbon, Lisbon, Portugal; 15 Lisbon, Portugal; 16 Department of Neurology, Charité Universitätsmedizin Berlin, Berlin, Germany; 17 University of Medicine Greifswald, Greifswald, Germany; 18 Department of Neurology and Neurosurgery, Institute of Clinical Medicine, Faculty of Medicine, Vilnius University, Vilnius, Lithuania; 19 Department of Neurology, Clinica Universidad de Navarra, Madrid, Spain; 20 Department of Neurology, Universitair Ziekenhuis Brussel (UZ Brussel), Brussels, Belgium; 21 Neuroprotection and Neuromodulation (NEUR) Research Group, Center for Neurosciences (C4N), Vrije Universiteit Brussel (VUB), Brussels, Belgium; 22 Division of Brain Sciences, Imperial College London, London, UK; 23 NorHead, Department of Neuromedicine and Movement Science, Norwegian University of Science and Technology (NTNU), Edvard Griegs gate, Trondheim, Norway

**Keywords:** Universal health coverage, Headache disorders, Migraine, Tension-type headache, Medication-overuse headache, Community pharmacists, Structured headache services, Global Campaign against Headache

## Abstract

In the sustainable development goals (SDG) context of seeking universal health coverage, the expanding gap between the supply of specialized and primary health-care providers of headache-related health care and the care needs of the very large number of people affected by headache is a formidable but not insoluble public-health challenge.

Structured headache services provide a cost-effective framework wherein controlled patient flows enable the care needs of people with headache to be met at appropriate levels, but these services may still be overwhelmed by inappropriate demand.

Community pharmacists are an underutilized resource, potentially well able to provide the solution. To do so, they must, as a profession, be integrated into structured headache services.

What remains to be determined is how to achieve this integration in an encouraging climate for change, which recognises the potential for relieving strained health-care systems and meeting a range of health-care needs by expanding pharmacists’ scope of practice.

This position statement on behalf of the European Headache Federation (EHF) and *Lifting The Burden* (LTB) is formally endorsed by the International Pharmaceutical Federation (FIP).

## Background

In the sustainable development goals (SDG) context of seeking universal health coverage (UHC) [1], the expanding gap between the supply and availability of health care for headache disorders and the health-care needs of the very large numbers of people affected by headache disorders is a formidable but not insoluble public-health challenge.

Headache disorders are the third highest cause of global ill health measured in years lived with disability (YLDs), accounting for 5.23% of all YLDs globally [2]. Migraine, a prevalent neurological disease affecting 14–15% of the world’s population [2, 3], alone accounts for 4.73% of global YLDs [2]. Tension-type headache (TTH) is even more prevalent, though less burdensome [2]. The societal costs of headache disorders are very high [4]. Despite this, throughout the world, headache is under-diagnosed and undertreated [5], a consequence of widespread health-care failure [6]. Everywhere, headache care is inadequately provided, and access to it is impeded by multiple barriers [6]. In the absence of care, reliance on over-the-counter (OTC) medications without guidance is a principal factor in causation of medication-overuse headache (MOH), a common and disabling – but avoidable – disease that adds substantially to the global burden of headache [7].

While many countries make little provision for headache care [6], the emphasis in those that do is often on specialized tertiary care, which is clinically and economically inefficient [5]. Most migraine and almost all TTH can be effectively managed in primary care, while primary care is best placed to recognise and forestall the development of MOH. Structured headache services based in primary care create and maintain links between primary, intermediate and specialist care (levels 1, 2 and 3), facilitating patients’ passage, while controlling flows, between these levels according to clinical need and available resources [5]. Economic evaluation has shown structured headache services to be cost-effective in all economies [8, 9].

But the more fundamental problem is one of capacity – because of the numbers involved (about 1.5 billion people worldwide [2]). A crucial premiss, on which structured headache services are predicated, is that some 50% of people with a headache disorder do not need recourse to medical services but, with advice and guidance, can adequately manage themselves [5]. The numbers dictate that they must do so in the interests of universal health coverage [1].

**Fig. 1:**
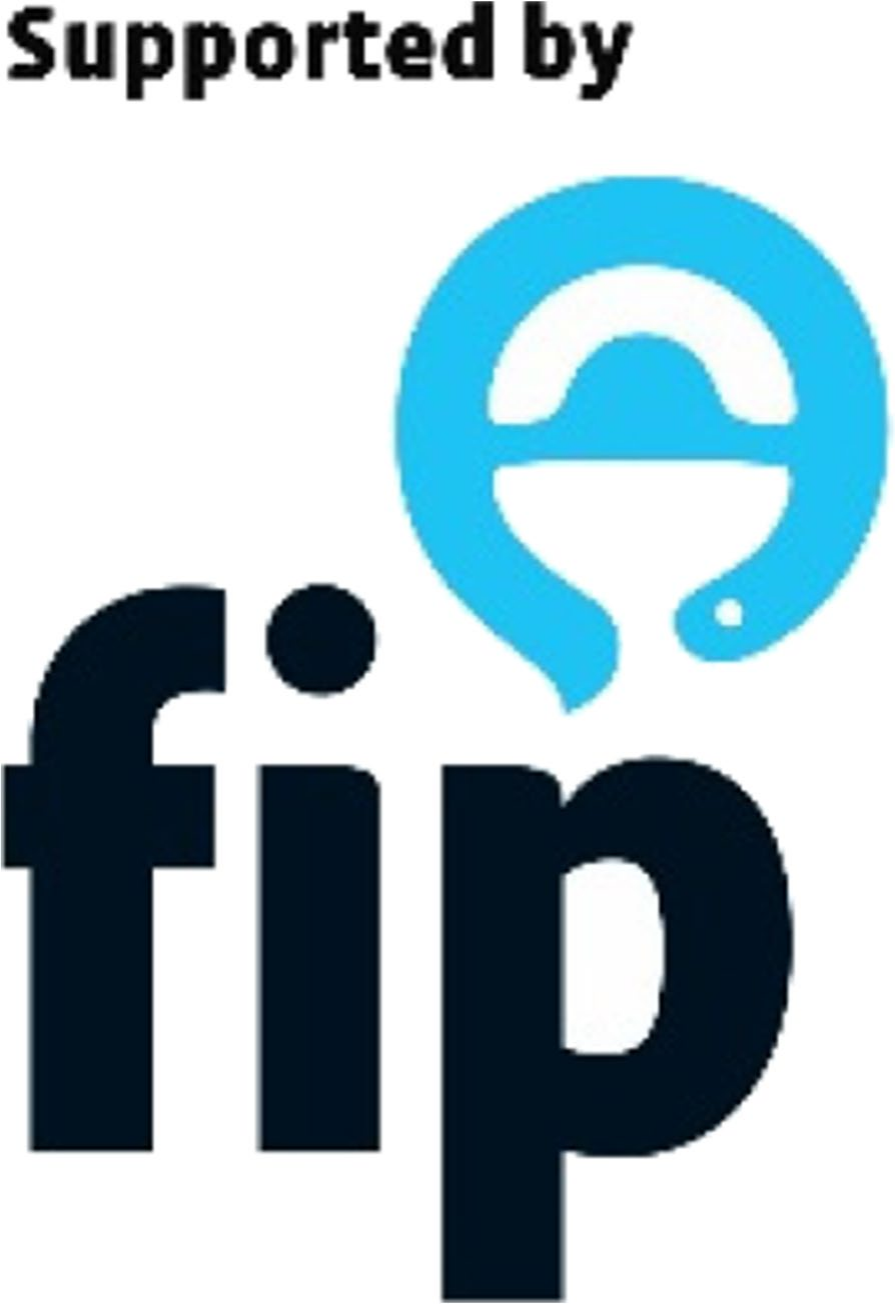
Formal endorsement by the International Pharmaceutical Federation representing multiple millions of pharmacists globally

This position statement on behalf of the European Headache Federation (EHF: the European scientific headache organisation [10]) and *Lifting The Burden* (LTB: the UK-registered non-governmental organisation conducting the Global Campaign against Headache in Official Relations with the World Health Organization [11, 12]) puts forward a solution, formally endorsed by the International Pharmaceutical Federation (FIP: the global body representing multiple millions of pharmacists [13]) (Fig 1). How to implement this solution is yet to be determined, but the way forward is proposed.

The purpose in publishing this statement is to stimulate interest, initiate debate and promote collaborative endeavour.

## Proposition

Community pharmacy services, a widely available but grossly underutilised resource in headache care, are strategically positioned for this advice and guidance role, which is already envisioned in the proposals for structured headache services [5]. Community pharmacists are the first port-of-call for people with troublesome headache in most of the world (setting aside traditional practitioners and herbalists). There is potential, currently being explored in several countries [14–16], for safely and effectively expanding pharmacists’ scope of practice.

Community pharmacists are health-care professionals expert in the effects, uses, misuses, interactions and contraindications of medication [16]. They are accessible at low cost and with no waiting-times, ideally placed and well able to provide guidance on self-management of headache [5]. Further, they can advise on when and how to seek medical care when this is needed, and they can contribute to community health education.

We propose that full integration of community pharmacists within structured headache services (level 0) is vital to the efficient operation of these services, and the only way to increase their capacity to meet need.

With basic understanding of headache, community pharmacists can underpin structured headache services in primary care and upwards, not only providing for the estimated 50% who do not need medical care (and those who cannot access medical care) but also reducing demand on over-burdened medical (primary and specialist) services [5].

We recognise that both public education and pharmacist education are among the necessary steps to achieve this integration.

### Argument and counterargument

Below we marshal the potential arguments against the proposition, and present counterarguments.

- *Migraine is a neurological disorder that can be difficult to diagnose, with no confirmatory tests. Headache, rarely, can be indicative of serious medical conditions. Pharmacists do not have the training to diagnose.*

Community pharmacists are not a replacement for medical care.

However, they are trained in a range of complex clinical functions to achieve therapy optimization [17, 18]. They have greatly superior knowledge to any that the average person with headache might have. They are entirely able to provide a competent and safe adjunctive and complementary health-care service [16, 18, 19].

With limited additional training, and provided with supportive management aids [20], pharmacists can recognize migraine, TTH, MOH and cluster headache, and, crucially, the red flags that may indicate serious headache (referring accordingly to medical services).

- *Community pharmacists’ scope of practice differs between countries.*

This is true, but differences in scopes of practice are not barriers that cannot be overcome by a global statement and local adaptations as needed. We argue for broadening the scope where necessary as a safe and cost-effective way forward, a process that is already beginning or under consideration in several countries in other therapeutic areas [14–19].

Counselling on medication usage, identifying problems, need for dosage adjustment, interactions and adverse effects, and assessing efficacy over time, are essential elements in optimization of therapy and part of all community pharmacists’ day-to-day work. MOH is a very good example of the consequences of lack of guidance in the correct use of medications [7, 20].

Counselling includes educating patients on the importance of adherence, the likely benefits of preventative medications if and only if adherence is good [21, 22], and on realistic expectations of treatment outcomes [20].

As an example of what is achievable, deployment of pharmacists around the globe in the SARS-CoV-2 (covid-19) pandemic took no account of availability of vaccines or treatment options but focused on accessibility [23].

- *The classification of medications as OTC or prescription-only differs between countries. The range of treatments pharmacists can provide is limited to the former.*

Expansion of pharmacists’ scope of practice to include prescribing from a limited list of drugs, safely and effectively for a range of common conditions, is being explored in some countries [14–16]. However, pharmacists have much to offer meanwhile.

OTC medications on WHO’s essential medicines list [24] are effective in over 50% of people with migraine or TTH [5, 20, 25–28]. Additionally, pharmacists can be provided with management aids and information leaflets [20].

In a recent study among pharmacists in Saudi Arabia, 83.7% believed people with migraine should try OTC before prescription medications [29]. In this and another study, > 80% of community pharmacists made up to five recommendations daily for OTC headache products [29, 30].

- *Community pharmacists may be reluctant to invest time in education and development of expertise in headache recognition and management.*

Pharmacists’ job satisfaction is negatively correlated with perceived helplessness, and positively correlated with self-efficacy beliefs [31]. Competence in the management of disorders that are common, and for which treatments are very frequently sought, is likely to have positive impact.

In two recent studies, 80% or more of community pharmacists felt that headache was an important or essential part of their practice [29, 30].

- *In many countries, paracetamol and NSAIDs are easily available from supermarket and local stores, without advice. Limitations on quantity, if in place, are easily evaded by visiting multiple outlets. Pharmacists have no means of controlling this.*

This is true. The solution lies in public education, in which community pharmacists can and should play a leading role. A key objective of public health education is to create awareness of pharmacists’ ready availability for expert advice.

- *Community pharmacists sell products. The business model behind provision of community pharmacy services in some countries may cause conflict between best-practice recommendations, including the expenditure of time in giving advice, and pharmacists’ own financial benefit.*

According to Deloitte [16]: “Today’s retail pharmacists are highly trained, trusted medical professionals who [in the US] spend a disproportionate amount of time counting pills and addressing clinical edits rather than operating at the top of their license (such as providing point-of-care testing and counseling).” This issue goes to the heart of the role and standing of community pharmacists as health-care professionals. It is not specific to these proposals, and on a general level is for pharmacists (and regulators) to resolve [16].

More broadly, conflict of interest arises from any billable service in health care, with, always, a legal duty of care to put patients’ interest first.

- *It is difficult to gauge the impact that community pharmacists might have on headache management.*

This can be overcome. In one example, a metanalysis reported that pharmacist-led medication review (performed either independently or as part of a multidisciplinary intervention) reduced chronic pain intensity, and improved physical functioning, quality of life and patient satisfaction [32]. In another, a United States study found that pharmacist-led risk assessment of opioid usage in a primary-care setting substantially improved provider adherence to guidelines and opioid prescribing practices [33].

## Way forward

The need for change is clear [1, 5], and the climate for change is encouraging [14–16, 18].

We propose the creation of a working group combining a range of competencies. Its tasks will be fourfold: a) to determine how the integration of community pharmacists within structured headache services might be achieved; b) to assess the willingness of community pharmacists in multiple countries to undertake this additional role; c) to assess the resources needed, including the educational requirements, and the economic viability of committing those resources; and d) to propose plans for practical implementation.

## Data Availability

No datasets were generated or analysed during the current study.

## Abbreviations

EHF: European Headache Federation
FIP: International Pharmaceutical Federation
LTB: *Lifting The Burden*
MOH: Medication-overuse headache
OTC: Over-the-counter
SDG: Sustainable development goals
TTH: Tension-type headache
UHC: Universal health coverage
YLD: Year lived with disability
