# Correction: The role of community pharmacists in managing common headache disorders, and their integration within structured headache services: position statement on behalf of the European Headache Federation (EHF) and *Lifting The Burden* (LTB: the Global Campaign against Headache), with the formal endorsement of the International Pharmaceutical federation

**DOI:** 10.1186/s10194-025-02073-5

**Published:** 2025-06-16

**Authors:** Heba BaniHani, Christian Lampl, Antoinette MaassenvandenBrink, Faisal Mohammad Amin, Louise Ninett Carlsen, Gianluca Coppola, Christina Deligianni, Raquel Gil‑Gouveia, Philip R. Holland, Andreas K. Husøy, Rigmor Jensen, Madalena Plácido, Uwe Reuter, Kristina Ryliškienė, Margarita Sanchez del Río, Henrik Winther Schytz, Erling Tronvik, Jan Versijpt, Timothy J. Steiner

**Affiliations:** 1 Norwegian Centre for Headache Research (NorHead), Trondheim, Norway; 2 Department of Neurology, Konventhospital Barmherzige Brüder, Linz, Austria; 3 Headache Medical Center Linz, Linz, Austria; 4 Department of Internal Medicine, Erasmus MC University Medical Center, Rotterdam, Netherlands; 5 Department of Neurology, Danish Headache Centre, Copenhagen University Hospital - Rigshospitalet, Copenhagen, Denmark; 6 Department of Medico‑Surgical Sciences and Biotechnologies, Sapienza University of Rome Polo Pontino ICOT, Latina, Italy; 7 Department of Neurology, AthensvNaval Hospital, Athens, Greece; 8 1st Neurology Department, Eginition Hospital, Medical School, National & Kapodistrian University of Athens, Athens, Greece; 9 Neurology Department, Hospital da Luz, Luz Saúde, Lisbon, Portugal; 10 Center for Interdisciplinary Research in Health, Universidade Católica Portuguesa, Lisbon, Portugal; 11 Headache Group, Wolfson Sensory Pain and Regeneration Centre, Institute of Psychiatry, Psychology and Neuroscience, King’s College London, London, UK; 12 Department of Neuromedicine and Movement Science, Norwegian University of Science and Technology (NTNU), Trondheim, Norway; 13 Department of Neurology and Clinical Neurophysiology, St Olavs University Hospital, Trondheim, Norway; 14 NOVA National School of Public Health, Public Health Research Centre, Comprehensive Health Research Centre, NOVA University of Lisbon, Lisbon, Portugal; 15 Lisbon, Portugal; 16 Department of Neurology, Charité Universitätsmedizin Berlin, Berlin, Germany; 17 University of Medicine Greifswald, Greifswald, Germany; 18 Department of Neurology and Neurosurgery, Institute of Clinical Medicine, Faculty of Medicine, Vilnius University, Vilnius, Lithuania; 19 Department of Neurology, Clinica Universidad de Navarra, Madrid, Spain; 20 Department of Neurology, Universitair Ziekenhuis Brussel (UZ Brussel), Brussels, Belgium; 21 Neuroprotection and Neuromodulation (NEUR) Research Group, Center for Neurosciences (C4N), Vrije Universiteit Brussel (VUB), Brussels, Belgium; 22 Division of Brain Sciences, Imperial College London, London, UK; 23 NorHead, Department of Neuromedicine and Movement Science, Norwegian University of Science and Technology (NTNU), Edvard Griegs gate, Trondheim, Norway

**Correction: J Headache Pain 26**,** 100 (2025)**

10.1186/s10194-025-02021-3

In this article, the text “on behalf of the European Headache Federation, the Norwegian Centre for Headache Research (NorHead) and Lifting The Burden: the Global Campaign against Headache” has been removed from the author list because it erroneously duplicated text from the article title.

